# Exploring air insole pressure and walking durations effects on microcirculation in healthy individuals to optimize diabetic foot ulcers prevention

**DOI:** 10.1038/s41598-025-94649-z

**Published:** 2025-03-27

**Authors:** Gilang Titah Ramadhan, Fahni Haris, Yih-Kuen Jan, Ben-Yi Liau, Wei-Cheng Shen, Jian-Guo Bau, Chun-Ming Lien, Chien-Cheng Tai, Chi-Wen Lung

**Affiliations:** 1 Department of Computer Science and Information Engineering, Asia University, Taichung, 413305 Taiwan; 2 School of Nursing, Universitas Muhammadiyah Yogyakarta, Yogyakarta, 55183 Indonesia; 3 Rehabilitation Engineering Lab, Department of Health and Kinesiology, University of Illinois at Urbana-Champaign, Urbana, IL 61801 USA; 4 Department of Automatic Control Engineering, Feng Chia University, Taichung, 407102 Taiwan; 5 Department of Creative Product Design, Asia University, Taichung, 413305 Taiwan; 6 Department of Agricultural Technology, National Formosa University, Yunlin, 632301 Taiwan; 7 Department of Commercial Design and Management, National Taipei University of Business, Taoyuan, 32462 Taiwan; 8 School of Public Health, Taipei Medical University, Taipei, 11031 Taiwan

**Keywords:** Blood flow, First metatarsal head, Peripheral vascular disease, Diabetic foot ulcers, Diabetes complications, Peripheral neuropathies

## Abstract

To evaluate the impact of air insole on reducing the risk of diabetic foot ulcers (DFUs) in healthy individuals through microcirculation assessment, which considers blood flow as a critical factor due to the role of peripheral vascular disease in DFU development. The study analyzes the interaction between air insole pressure and walking duration. Repeated-measures design was used to assess the effects of two walking durations (10 and 20 min) and three air insole pressures (80, 160, and 240 mmHg), resulting in six walking conditions tested in 13 healthy participants. The average blood flow in the first metatarsal head (M1) was quantified using data from the last 3 min of the 10 min post-exercise period. The results of one-way ANOVA showed that the 80 mmHg air insole pressure was significantly lower microcirculation than 240 mmHg with 10 min walking duration (129.4 ± 9.1 vs 163.1 ± 12.6 PU, *P* = 0.035). The paired t-test showed three significant differences in the effects of the walking duration in all air insole pressures. (1) 10 min walking duration was significantly lower compared to 20 min with 80 mmHg air insole pressure (129.4 ± 9.1 vs 203.6 ± 10.1 PU, *P* = 0.001); (2) 10 min walking duration was significantly lower compared to 20 min with 160 mmHg air insole pressure (142.5 ± 10.6 vs 206.0 ± 12.5 PU, *P* = 0.001); (3) 10 min walking duration was significantly lower compared to 20 min with 240 mmHg (163.1 ± 12.6 vs 219.1 ± 11.8 PU, *P* = 0.008). This study highlights that walking with an air insole pressure of 80 mmHg for 20 minutes influences microcirculation at the first metatarsal head, potentially offering important benefits for individuals at risk of pressure-related injuries, such as DFUs.

## Introduction

The long-term presence of diabetes mellitus (DM) may lead to the development of complications involving various pathological changes such as peripheral neuropathy, vasculopathy, and foot deformities, which may lead to diabetic foot ulcers (DFUs)^1^. In 2022, the total estimated cost of diagnosed DM in the U.S. was $412.9 billion, comprising $306.6 billion in direct medical costs and $106.3 billion in indirect costs^2^. Diabetes-related expenses account for 25% of all healthcare spending in the U.S., and individuals with diabetes face medical costs approximately 2.6 times higher than those without the disease^2^. Given the substantial medical costs and high occurrence of DFUs, the importance of DFU prevention is emphasized in diabetes management^3^.

Many studies commonly use peak plantar pressure (PPP) to predict the risk of DFUs based on mechanical stress on the foot by measuring the highest pressures during walking, providing insight into areas at risk for ulceration due to pressure-related damage^4,5^. In comparison, microcirculation provides insights into vascular components. Comparing microcirculation and PPP in predicting DFUs revealed that both methods are valuable. Microcirculation has distinct advantages since it focuses more on the vascular component by assessing blood flow in small vessels, thereby identifying possible vascular complications contributing to ulcer development^6^. Microcirculation provides a clearer view of the vascular condition and potential for healing, which is critical because peripheral vascular disease significantly contributes to DFUs^7^. Therefore, assessing microcirculation can be more informative for preventing and managing DFUs than relying solely on mechanical measures, such as PPP. Importantly, diabetes-related microvascular and macrovascular diseases are independent processes, and targeted microcirculatory assessments provide essential insights into diabetic microvascular complications, improvements in small vessel perfusion can significantly influence healing outcomes, offering valuable guidance for DFU management and prevention^8^.

Exercise could be used as a preventive intervention for people with diabetes, promoting health and reducing the risk of chronic complications associated with DM^9^. Walking is the most prevalent form of exercise among individuals with DM^10,11^. According to the latest guidelines from the International Working Group on the Diabetic Foot (IWGDF), patients with low or moderate risk of foot ulcers are encouraged to engage in foot-related exercises and increase weight-bearing activity, such as walking an additional 1000 steps daily, to reduce the incidence of DFUs among this at-risk population^12^.

Walking affects microcirculation by enhancing blood flow in smaller vessels, which can help deliver oxygen and nutrients to tissues^13^. This happens because walking increases systemic wall shear stress, increasing plasma nitrite concentrations due to increased endothelial nitric oxide synthase (eNOS) activity, which regulates blood flow^14^. Increasing the microcirculation, particularly the tissue oxygen saturation and capillary blood flow, can promote wound healing^15^. However, excessive or improper loading can disrupt the optimal microcirculation response of the foot during walking^16^. Variation in the hardness levels of insoles is an important factor in effectively redistributing pressure and also influences microcirculation, as has been stated by Haris et al. and Duan et al.^5,7^. When pressure from insoles is suitable during walking, it promotes optimal microcirculation by evenly distributing pressure across the foot, enhancing comfort, and preventing vascular issues^17^. Thus, choosing insoles with the appropriate hardness is crucial for maintaining healthy microcirculation and foot health.

In addition, Walking continuously for a long period can also lead to fatigue and strain on the cardiovascular system^18^. Therefore, longer walking duration and an increased complexity index of the center of pressure (COP) may change the structure and function of the soft tissues refers to the variability and adaptability of the COP patterns under different conditions, which can increase the risk of developing DFUs^19^. Short-duration walking can also benefit by providing sufficient stimulus to improve microcirculation without causing excessive strain, but the benefits might be less pronounced than longer durations^20^. There is a risk of overexertion with prolonged walking, leading to vascular and muscular fatigue, while short durations may not provide sufficient stimulus for optimal cardiovascular benefits^21^. Previous studies have reported that walking with various durations and air insole pressure can affectfoot properties^22,23^. Determining the appropriate walking duration is important for maintaining health and preventing DFUs.

The results of this study in healthy participants can optimize the air insole design, which provides a foundation for understanding the effect of air insole pressure and walking duration on microcirculation in healthy individuals to prevent DFU. This study’s use of healthy participants is justified as it aims to establish baseline data and control conditions free from the confounding variables associated with DFUs. Research shows that interventions to prevent foot ulcers benefit from a robust understanding of plantar biomechanics and pressure distribution. This can be effectively studied in healthy participants before being applied to those at risk of DFUs^12^. Furthermore, studies have shown that biomechanical analyses do not always require non-insole baseline data, as interventions can be effectively evaluated through controlled, comparative conditions^24^. We hypothesized that the effect of different air insole pressures and walking durations may result in microcirculation suitable for mitigating the risk of DFUs. Therefore, this study aimed to determine which walking condition would be suitable based on the effect of air insole pressure and walking duration on microcirculation to reduce the risk of DFUs.

## Methods

This study used a repeated-measures design to assess the effects of two walking durations (10 and 20 min) and three air insole pressures (80, 160, and 240 mmHg), which refers specifically to the study design where the same participants are measured repeatedly^25^. According to a previous study, a GS-701N Shore durometer (Teclock Co., Ltd., Nagano, Japan) was used to determine the hardness values of the air insole^26^. An air pump and pressure gauge were used to fill the air insole to the desired pressures that cover the forefoot to specifically target and alleviate the high plantar pressures and shear forces concentrated in the metatarsalgia region, especially the first metatarsal head (M1), which are critical factors in the development of foot ulcers and other forefoot-related conditions. By focusing on the forefoot, air insoles can relieve localized pressure and improve shock absorption^27^. In our earlier study, air insole pressures were selected to ensure appropriate stiffness for walking^5^.

### Participants

The study included 13 healthy participants (7 men and 6 women) aged 21–39 years, with a mean (± SD) age of 27.0 ± 7.3 years, body weight of 56.0 ± 7.9 kg, height of 165.8 ± 8.4 cm, and BMI of 20.3 ± 1.7 kg/m^2^. This study was approved by the Central Regional Research Ethics Committee China Medical University, Taichung, Taiwan, under the reference number CRREC-111-017. Before participating in the study, all participants received information about the study’s purpose and procedures. Written consent was obtained from all participants, who were assured that their information would remain confidential. Following the Declaration of Helsinki, the respondents were allowed to withdraw from the study at any time, and their responses were anonymized. To be eligible to participate, participants must be at least 18 years old, wear shoe sizes 41 to 42 (males) or 37 to 39 (females), and have a body mass index (BMI) of no more than 23.0 kg/m^2^. The restriction on shoe sizes helps ensure consistent results by reducing variability in foot mechanics and comfort, as improper shoe size can affect gait and pressure^28^. Similarly, limiting BMI creates a more uniform group, making it easier to study interventions or correlations without extra adjustments, while focusing on variables like blood pressure and recent foot health^29^. All subjects with active foot ulcers, diabetes, vascular disease, hypertension, and the inability to walk for 20 min independently were not included in the study^5^.

All examinations were performed in the Biodesign Lab at the Asia University, Taichung, Taiwan. The room temperature was maintained at 24 ± 2 °C. Exposure to physical or environmental stressors may challenge microvascular regulation, as Litvin describes, because these stressors require the body to adapt to maintain blood flow, often complicating the stability of microvascular measurements^30^. In addition, factors beyond the direct study variables can significantly influence microvascular measurements. For instance, caffeine intake reduces microcirculation due to its vasoconstrictive effects, which limit blood flow in smaller vessels^31^. Nicotine increases microcirculation due to its stimulatory effect on the cardiovascular system, causing increased blood flow and microvascular response^32^. These variables are carefully controlled to ensure accurate and reliable microvascular assessments.

### Experimental procedure

The participants were instructed to take off their socks and shoes and lie down for 20 min to minimize the impact of prior weight-bearing activities on muscle fatigue and plantar pressure. The specific walking procedure was as follows: (1) 80 mmHg air insole pressure with a 10 min walking duration; (2) 80 mmHg air insole pressure with a 20 min walking duration; (3) 160 mmHg air insole pressure with a 10 min walking duration; (4) 160 mmHg air insole pressure with a 20 min walking duration; (5) 240 mmHg air insole pressure with a 10 min walking duration; (6) 240 mmHg air insole pressure with a 20 min walking duration.

**Fig. 1:**
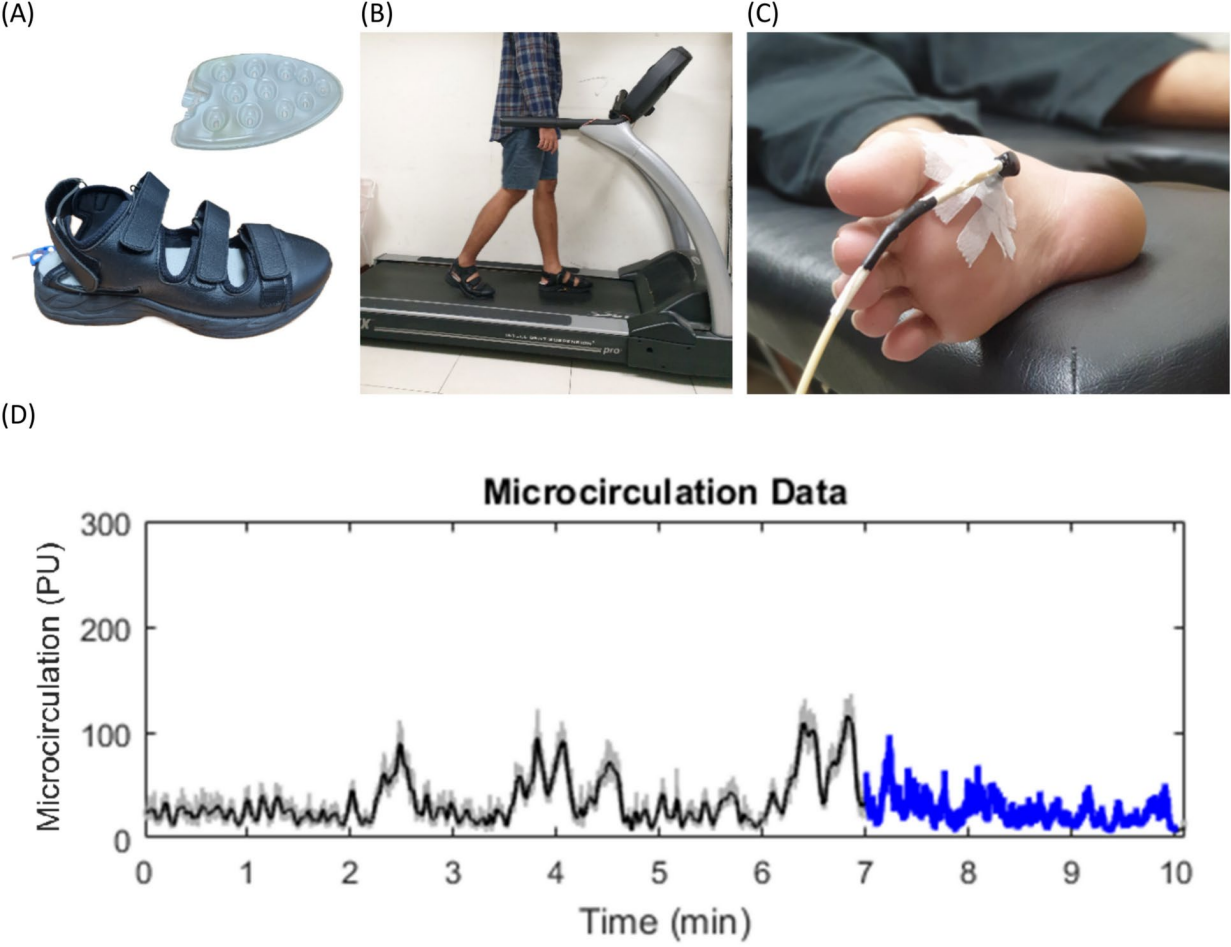
Retrieval of microcirculation data from M1. (**A**) Shoes with air insole; (**B**) Participants walk on a treadmill; (**C**) Retrieval of microcirculation data on M1; (**D**) Preview Microcirculation Data with an average of last 3 min from 10 min, measured in PU, perfusion units.

Before data collection, participants could choose the order in which they completed the three air insole pressures and two walking durations based on their preference. This flexible approach ensured that the order of conditions did not influence the outcomes, ensuring unbiased data collection guided by each participant’s choice^22,23^. To minimize the likelihood of carryover effects when participants conducted the test twice a day, a washout period of 20 min was implemented. Participants were instructed to wear commercial shoes (Hsin He Hsin Co., Ltd., Taichung, Taiwan) with air insole pressure, whose hardness was adjusted as shown in Fig. 1A. The participants were then asked to walk on a treadmill (Cybex DE-20427 A, manufactured by Cybex in Taoyuan, Taiwan) with a walking speed of 3.6 mph as illustrated in Fig. 1B. According to the American Diabetes Association and the American Guidelines, a walking speed of 3.6 mph was considered common^33,34^. The microvascular blood flow and oxygenation dynamics were quantified using a laser Doppler flowmeter (Moor-VMS-LDF2, Moor Instruments Ltd., Axminster, UK) to measure blood flow.

Microcirculation data were collected before and after the exercise by having participants lie in the designated area in a relaxed position. The fiber optic probe of laser Doppler flowmeter was positioned on the M1 to retrieve the microcirculation data, as illustrated in Fig. 1C. A key factor in selecting this location was the significance of the blood supply to M1, which is frequently involved in foot ulceration^35^. Participants were given three or five minutes to walk on a treadmill to familiarize themselves with the shoes before stepping on one^36,37^.

### Data analysis

The microcirculation data values were obtained by averaging the last 3 min of the microcirculation values from a 10 min post-exercise period, as illustrated in Fig. 1D. Microcirculation measurements typically require an initial period for flux values to stabilize. The first several minutes may show fluctuations as the system responds to initial conditions or the measurement setup. By focusing on the last 3 min, we capture more stable data that reflects the subject’s baseline after these initial variations subside^38^. All data were analyzed using MATLAB 2022b (The MathWorks, Natick, MA, USA). The data were filtered using an 8th Butterworth low-pass filter with a sampling rate of 40 Hz. The microcirculation data were determined from the average 3 min microcirculation in M1 using Eq. (1):

1 $$Microcirculation= \frac{\sum Xi}{n}$$

***Xi*** is the microcirculation at the ***i-th*** frequency, and ***n*** is the total number of frames in frequency.

### Statistical analysis

The microcirculation values are presented as mean ± standard error. One-way analysis of variance (ANOVA) with the least significant difference (LSD) post hoc test was used to analyze the effect of the air insole pressure on microcirculation^5^. In addition, a paired t-test was used to analyze the effect of walking duration on microcirculation^23^. To further assess the magnitude of the observed effects, effect sizes were calculated using Cohen’s d, with 95% confidence intervals (95% CI) provided to quantify the precision of the estimates and enhance the interpretability of the results. All statistical analyses were performed using SPSS version 22 (IBM, NY, USA) with a significance level of 0.05. A sample size calculation was not conducted for this study, as the sample was selected based on convenience. This limitation should be considered when interpreting the results, as it may affect the generalizability of the findings. However, small sample sizes are sometimes justified in preliminary or exploratory studies. For instance, Julious (2005) notes that a sample size of around 12 per group can suffice in early trials or pilot studies, especially when there is limited prior data^39^.

## Result

A total of 13 healthy participants (seven men and six women) aged 21 to 39 years were enrolled in this study, with shoe sizes of 41–43 for men and 36–38 for women, body weights less than 80 kg, and dominance of the right leg. As a result, the characteristics of participants (mean ± SD) were as follows: age 27.9 ± 7.7 years, weight 56.9 ± 7.9 kg, height 165.8 ± 8.4 cm, and body mass index 20.3 ± 1.7 kg/m^2^. Measurements obtained at various pressures showed values of 51.7 ± 1.5 Shore at 80 mmHg, 54.7 ± 0.6 Shore at 160 mmHg, and 57.7 ± 0.6 Shore at 240 mmHg.

**Fig. 2:**
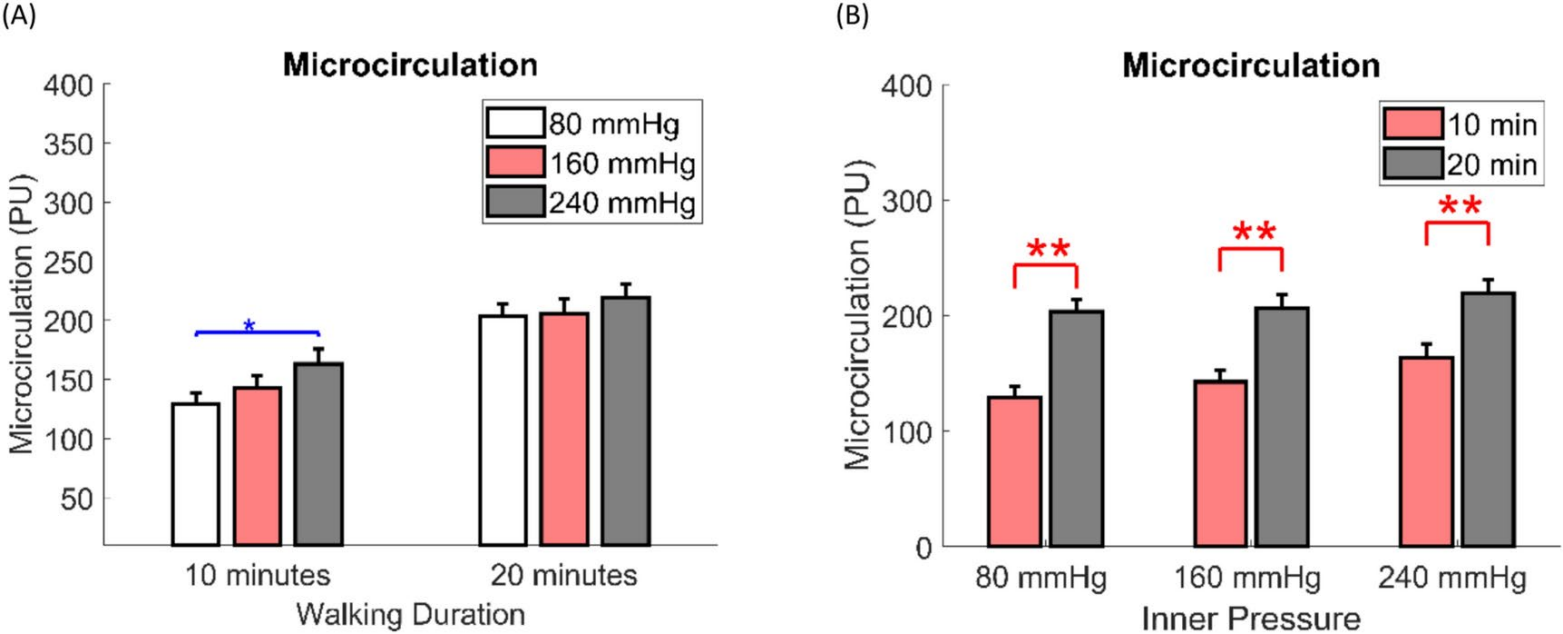
Bar chart comparisons (**A**) Comparisons of the effect of the walking duration on the microcirculation at 3 air insole pressure of air insoles. (**B**) Comparisons of the effect of the air insole pressure on the microcirculation at 2 walking durations. Data are shown as mean ± standard errors. *A significant difference (*P* < 0.05) and **A significant difference (*P* < 0.01). *PU* perfusion units.

The results of the one-way ANOVA indicated that microcirculation at an 80 mmHg air insole pressure was significantly lower than at 240 mmHg during a 10 min walking duration (Table 1, Fig. 2A).

**Table 1:** Effect of air insole pressure on the microcirculation.

Region	Walking duration	Air insole pressure			One-way	LSD		
					ANOVA	Post hoc		
		80 mmHg (Mean ± SE)	160 mmHg (Mean ± SE)	240 mmHg (Mean ± SE)	*P* value	80 mmHg vs	80 mmHg vs	160 mmHg vs
						160 mmHg	240 mmHg	240 mmHg
M1 (PU)	10 min	129.4 ± 9.1	142.5 ± 10.6	163.1 ± 12.6	0.101	0.400	0.035*	0.188
	20 min	203.6 ± 10.1	206.0 ± 12.5	219.1 ± 11.8	0.596	0.884	0.348	0.426

Data are presented as mean ± standard error; *Significant difference (*P* < 0.05). *PU* perfusion units.

Additionally, the paired t-test revealed significant differences in the effects of walking duration. Specifically, the 10 min walking duration was significantly lower than the 20 min duration at 80 mmHg air insole pressure; the same trend was observed at 160 mmHg and 240 mmHg air insole pressures (Table 2, Fig. 2B).

**Table 2:** Effect of walking duration on the microcirculation.

Parameter	Air insole pressure	Walking duration		Paired t-test
		10 min (Mean ± SE)	20 min (Mean ± SE)	*P* value
M1 (PU)	80 mmHg	129.4 ± 9.1	203.6 ± 10.1	0.001**
	160 mmHg	142.5 ± 10.6	206.0 ± 12.5	0.001**
	240 mmHg	163.1 ± 12.6	219.1 ± 11.8	0.008**

Data are the mean ± standard error; **A significant difference (*P* < 0.01). *PU* perfusion units.

In this study, the mean difference in microcirculation measurements will be used as a variable to calculate the effect size using Cohen’s d formula. This statistical method quantifies the magnitude of the difference between groups by dividing the mean difference by the pooled standard deviation. Calculating Cohen’s d will provide a standardized measure of the effect size, allowing us to assess the practical significance of walking duration and insole inner pressure on microcirculatory changes using Eq. (2):

2 $$d= \frac{Mean \, difference}{Standard \, Deviation \, of \, Difference}$$

The analysis revealed that 20-min walking durations consistently demonstrated improved microvascular perfusion compared to 10-min walking durations. This improvement was more pronounced at lower insole inner pressures, which showed higher mean differences. The comparison of 80 mmHg with 10 min and 80 mmHg with 20 min revealed a large effect size (d = 2.14). Similarly, the comparison between 160 mmHg with 10 min and 160 mmHg with 20 min showed a large effect size (d = 1.52). Finally, the comparison between 240 mmHg with 10 min and 240 mmHg with 20 min yielded a large effect size (d = 1.28). Table 3 summarizes the effects of walking duration on microcirculation mean differences, including the corresponding effect sizes (Cohen’s d) and 95% CI for the mean differences.

**Table 3:** Effect of walking duration on microcirculation mean difference.

Comparison	Mean difference	Effect size (Cohen’s d)	95% CI
80 mmHg with 10 min vs. 80 mmHg with 20 min	− 74.2	2.14	− 98.38 to − 50.08
160 mmHg with 10 min vs. 160 mmHg with 20 min	− 63.5	1.52	− 96.47 to − 30.61
240 mmHg with 10 min vs. 240 mmHg with 20 min	− 56.0	1.28	− 94.77 to − 17.22

*CI* Confidence Intervals.

Similarly, the analysis effect of insole inner pressure revealed that lower insole pressures consistently resulted in lower mean differences compared to moderate and higher pressures for both 10- and 20 min walking durations. The comparison of 10 min with 80 mmHg and 10 min with 160 mmHg revealed a small effect size (*d* = 0.37). The comparison between 10 min with 80 mmHg and 10 min with 240 mmHg showed a moderate effect size (*d* = 0.85). Similarly, the comparison between 10 min with 160 mmHg and 10 min with 240 mmHg yielded a moderate effect size (*d* = 0.49). The comparison of 20 min with 80 mmHg and 20 min with 160 mmHg revealed a negligible effect size (*d* = 0.06). The comparison between 20 min with 80 mmHg and 20 min with 240 mmHg showed a small effect size (*d* = 0.39). Similarly, the comparison between 10 min with 160 mmHg and 10 min with 240 mmHg yielded a small effect size (*d* = 0.30). Table 4 provides a detailed summary of these findings.

**Table 4:** Effect of insole inner pressure on microcirculation mean difference.

Mean	Mean difference	Effect size (Cohen’s d)	95% CI
10 min with 80 mmHg vs. 10 min with 160 mmHg	− 13.08	0.37	− 44.21 to 18.06
10 min with 80 mmHg vs. 10 min with 240 mmHg	− 33.69	0.85	− 64.83 to − 2.55
10 min with 160 mmHg vs. 10 min with 240 mmHg	− 20.62	0.49	− 51.75 to 10.52
20 min with 80 mmHg vs. 20 min with 160 mmHg	− 2.38	0.06	− 35.36 to 30.59
20 min with 80 mmHg vs. 20 min with 240 mmHg	− 15.46	0.39	− 48.43 to 17.51
20 min with 160 mmHg vs 20 min with 240 mmHg	− 13.08	0.30	− 46.05 to 19.89

*CI* Confidence Intervals.

**Fig. 3:**
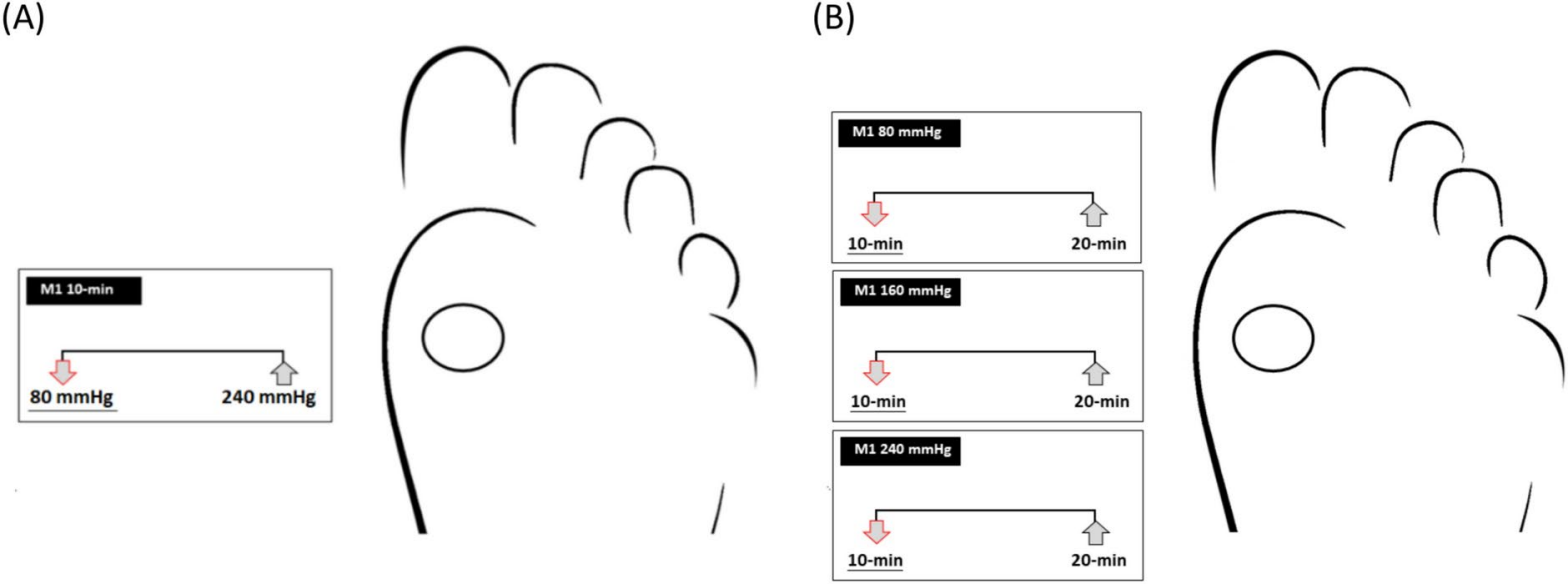
Illustration of the effect of air insole pressure and walking duration on the microcirculation (**A**) The effect of the air insole pressure showed 80 mmHg, which was lower microcirculation than 240 mmHg at 10 min. (**B**) The effect of walking durations on the microcirculation showed that 80, 160, and 240 mmHg with 10 min were lower than 20 min.

This study’s results determined the effect of the air insole pressure on the foot microcirculation. Walking with 10 min (shorter durations) and 80 mmHg air insole pressure resulted in a significantly lower microcirculation than walking with shorter durations and 240 mmHg air insole pressure illustrated in Fig. 3A. Furthermore, walking for a shorter duration produced significantly lower microcirculation than walking longer at all air insole pressures illustrated in Fig. 3B.

## Discussion

Compared to 240 mmHg air insole pressure, walking with 80 mmHg air insole pressure significantly decreases microcirculation at the M1. However, while 80 mmHg air insole pressure still produces lower microcirculation over a longer duration than 240 mmHg air insole pressure, the difference is no longer statistically significant. This is believed since 80 mmHg air insole pressure offers better cushioning and distributes pressure evenly across the foot, reducing localized stress that may lead to reduced blood flow and microcirculation in specific areas like the M1^40,41^. Lower PPP is also beneficial for preventing DFUs as it minimizes the risk of tissue damage caused by prolonged pressure^42^.

High microcirculation benefits healthy individuals by supporting sufficient blood flow and tissue oxygenation. It is essential in preventing DFUs by maintaining tissue viability and reducing the risk of infection^43^. In individuals with diabetes, impaired microvascular function reduces the ability to respond to tissue stress, such as injury or infection. Improving microcirculation is vital for enhancing oxygen delivery, nutrient supply, and waste removal, all of which are essential for effective wound healing^44^. It is crucial to improve microcirculation after walking to prevent DFUs because diabetic feet typically exhibit thickened basement membranes, which can hinder leukocyte movement and increase hyperemic reactions, increasing the risk of infection^45^.

Additionally, reduced capillary blood flow in diabetic feet indicates maldistribution and functional skin ischemia, leading to diminished skin microvascular flow and reduced maximum hyperemic response to thermal stress^46^. This results in microvascular dysfunction and an inability to vasodilate and achieve optimal blood flow, making it one of the key microcirculatory factors contributing to DFUs. Moreover, impaired microvascular reactivity after walking on a diabetic foot has been implicated as the main contributing factor to foot ischemia in diabetes^35^. Adequate microcirculation is essential for tissue health, ensuring oxygen is delivered to the skin and underlying tissues^15^. When impaired microcirculation, the reduced blood flow can lead to tissue hypoxia and poor wound healing, increasing the risk of ulcers and infections^47^.

As a result of our findings, we found that adding walking duration can significantly increase microcirculation. Additionally, according to previous research conducted by Haris, increasing walking duration did not significantly affect PPP on M1. Therefore, increasing walking duration could be considered safe^5^. A longer walking duration can significantly improve microcirculation by increasing blood flow to tissues and oxygen delivery, thereby improving vascular health.

After walking for a longer duration with various air insole pressures, there was an improvement in peripheral vascular resistance, which might be the reason for the faster oxygen reperfusion rate and improvement in vascular reactivity^48^. In addition, one of the causes of the significant increase in microcirculation when walking longer with different air insole pressure is the skin temperature factor. When the foot temperature gradually rises as a result of exercise, the duration of the exercise also increases^49^. It is widely known that as the temperature increases, vasodilation in the skin may be induced, which in turn leads to an increase in blood flow.

In conjunction with the rise in temperature, the permeability of potassium in the smooth muscles surrounding endothelial cells also increases^50^. Therefore, the smooth muscles of the vascular system can relax, improving blood perfusion regardless of the air insole pressure used^51,52^. When individuals are at higher risk for developing DFUs, walking with 80 mmHg air insole pressure and longer walking duration can improve overall comfort and contribute to better foot health even though walking using 80 mmHg and 240 mmHg air insole pressure shows higher microcirculation. Thus, choosing appropriate air insole pressure can be a crucial preventive measure for foot health, promoting comfort while reducing the potential for ulcers.

However, this study had some limitations. First, the study only measured the immediate effects of different air insole pressures and walking durations on microcirculation without considering any delayed outcomes. Other research suggests that walking under various insole conditions might influence microcirculation not immediately but after a few hours or even days, indicating potential delayed responses. The delayed response may be due to the time required for these physiological adaptations to manifest fully, which is critical when red blood cell deformability and aggregation significantly impact blood flow dynamics^53^. Second, although meticulously controlled, the experimental setup did not fully capture the complexity of real-world walking conditions and the diverse environments typically experienced by individuals. The study setting didn’t have a walking field similar to real life and footwear variability, which can significantly influence microcirculatory responses, leading to different outcomes that may not reflect everyday scenarios^54^. Third, this study’s sample size of 13 participants is notably small, impacting statistical power and potentially the robustness of findings. The last limitation is the study’s restriction to the shoe size limits and BMI’s generalizability of the findings, as differences in shoe size or BMI may significantly affect outcomes in footwear-related research^55^. Variation is essential to ensure results are applicable across diverse populations, emphasizing the critical impact of proper shoe size for accurate research in foot health and mobility settings^56^. There is a need for further studies to confirm these findings and evaluate the long-term clinical outcomes of these insoles in larger populations, more diverse walking fields and a broader range of shoe sizes and foot dimensions.

## Conclusion

This investigation underscores the vital influence of air insole pressure and walking duration on foot microcirculation. A key takeaway from our findings is the association between walking with an air insole pressure of 80 mmHg for a 20 min duration and changes in microcirculation at the first metatarsal head. These findings may have important implications for individuals at risk of pressure-related injuries, such as DFUs. Our research provides valuable evidence for implementing targeted walking protocols to reduce DFU risk. By focusing on the interplay between air insole pressure and walking duration, we see significant potential for optimizing footwear interventions for healthy individuals, ultimately improving foot health and preventing ulcerations.

## Data Availability

Data is provided within the manuscript.

## References

[1] Gnanasundaram, S. et al. Gait changes in persons with diabetes: Early risk marker for diabetic foot ulcer. *Foot Ankle Surg.* **26**(2), 163–168 (2020). 30712991 10.1016/j.fas.2019.01.005

[2] Parker, E. D. et al. Economic costs of diabetes in the US in 2022. *Diabetes Care* **47**(1), 26–43 (2023). 10.2337/dci23-0085 37909353

[3] Liao, F. et al. Effect of exercise on risk factors of diabetic foot ulcers: A systematic review and meta-analysis. *Am. J. Phys. Med. Rehabil.* **98**(2), 103–116 (2019). 30020090 10.1097/PHM.0000000000001002

[4] Haris, F. et al. A review of the plantar pressure distribution effects from insole materials and at different walking speeds. *Appl. Sci.* **11**(24), 11851 (2021).

[5] Haris, F. et al. The effects of different inner pressures of air insoles and walking durations on peak plantar pressure. *Medicine* **102**(43), e35704 (2023). 37904356 10.1097/MD.0000000000035704 PMC10615489

[6] Neubauer-Geryk, J. et al. Current methods for the assessment of skin microcirculation: Part 1. *Postepy Dermatol. Alergol.* **36**(3), 247–254 (2019). 31333339 10.5114/ada.2019.83656 PMC6640017

[7] Duan, Y. et al. The effects of different accumulated pressure-time integral stimuli on plantar blood flow in people with diabetes mellitus. *BMC Musculoskeletal Disord.* **22**(1), 554 (2021). 10.1186/s12891-021-04437-9 PMC8214278 34144680

[8] Hilty, M. P. et al. Assessment of endothelial cell function and physiological microcirculatory reserve by video microscopy using a topical acetylcholine and nitroglycerin challenge. *Intensive Care Med. Exp.* **5**(1), 26 (2017). 28523563 10.1186/s40635-017-0139-0 PMC5436993

[9] Wu, F. L. et al. Microvascular control mechanism of the plantar foot in response to different walking speeds and durations: Implication for the prevention of foot ulcers. *Int. J. Low Extrem. Wounds* **20**(4), 327–336 (2021). 32326799 10.1177/1534734620915360

[10] Wu, F. L. et al. Effects of walking speeds and durations on plantar skin blood flow responses. *Microvasc. Res.* **128**, 103936 (2020). 31670165 10.1016/j.mvr.2019.103936

[11] Lung, C. W. et al. Effects of various walking intensities on leg muscle fatigue and plantar pressure distributions. *BMC Musculoskeletal Disord.* **22**(1), 4705 (2021). 10.1186/s12891-021-04705-8 PMC8477480 34579699

[12] Bus, S. A. et al. Guidelines on the prevention of foot ulcers in persons with diabetes (IWGDF 2023 update). *Diabetes Metab. Res. Rev.* **40**(3), e3651 (2024). 37302121 10.1002/dmrr.3651

[13] McDermott, M. M. et al. Walking exercise therapy effects on lower extremity skeletal muscle in peripheral artery disease. *Circ. Res.* **128**(12), 1851–1867 (2021). 34110902 10.1161/CIRCRESAHA.121.318242

[14] Muskat, J. C. et al. Transport of nitrite from large arteries modulates regional blood flow during stress and exercise. *Front. Cardiovasc. Med.* **10**, 1146717 (2023). 37378407 10.3389/fcvm.2023.1146717 PMC10291090

[15] Jensen, J.-O. et al. The repetitive application of cold atmospheric plasma (CAP) improves microcirculation parameters in chronic wounds. *Microvasc. Res.* **138**, 104220 (2021). 34216601 10.1016/j.mvr.2021.104220

[16] Zhang, Z. et al. Dynamic microcirculation characteristics of plantar skin under metatarsal head of human foot in response to life-like pressure stimulus. *Microcirculation* **31**, e12860 (2024). 38837938 10.1111/micc.12860

[17] Peng, H.-T. et al. The soft prefabricated orthopedic insole decreases plantar pressure during uphill walking with heavy load carriage. *Bioengineering* **10**(3), 353 (2023). 36978744 10.3390/bioengineering10030353 PMC10045236

[18] Prasertsri, P. et al. Effects of long-term regular continuous and intermittent walking on oxidative stress, metabolic profile, heart rate variability, and blood pressure in older adults with hypertension. *J. Environ. Public Health* **2022**(1), 5942947 (2022). 35140794 10.1155/2022/5942947 PMC8820939

[19] Liau, B. Y. et al. Using bidimensional multiscale entropy analysis of ultrasound images to assess the effect of various walking intensities on plantar soft tissues. *Entropy* **23**(3), 264 (2021). 33668190 10.3390/e23030264 PMC7995977

[20] Murphy, M. H. & Hardman, A. E. Training effects of short and long bouts of brisk walking in sedentary women. *Med. Sci. Sports Exerc.* **30**(1), 152–157 (1998). 9475657 10.1097/00005768-199801000-00021

[21] Quinn, T. J., Vroman, N. B. & Kertzer, R. Postexercise oxygen consumption in trained females: effect of exercise duration. *Med. Sci. Sports Exerc.* **26**(7), 908–913 (1994). 7934767

[22] Ramadhan, G. T. et al. Effect of different inner pressures of air insoles and walking durations on plantar pressure time integral. *Sci. Rep.* **14**(1), 19272 (2024). 39164374 10.1038/s41598-024-70312-x PMC11336220

[23] Haris, F. et al. Plantar pressure gradient and pressure gradient angle are affected by inner pressure of air insole. *Front. Bioeng. Biotechnol.* **12**, 1353888 (2024). 38529404 10.3389/fbioe.2024.1353888 PMC10961410

[24] Koska, D., Oriwol, D. & Maiwald, C. Comparison of statistical models for characterizing continuous differences between two biomechanical measurement systems. *J. Biomech.* **149**, 111506 (2023). 36806004 10.1016/j.jbiomech.2023.111506

[25] Dixon, P. Models of accuracy in repeated-measures designs. *J. Mem. Lang.* **59**(4), 447–456 (2008).

[26] Helili, M. et al. An investigation of regional plantar soft tissue hardness and its potential correlation with plantar pressure distribution in healthy adults. *Appl. Bion. Biomech.* **2021**, 5566036 (2021). 10.1155/2021/5566036 PMC8241530 34239603

[27] Ahmed, S. et al. Footwear and insole design features that reduce neuropathic plantar forefoot ulcer risk in people with diabetes: A systematic literature review. *J. Foot Ankle Res.* **13**(1), 30 (2020). 32498719 10.1186/s13047-020-00400-4 PMC7271493

[28] Hayashi, R. & Hosoya, S. Effect of improperly sized shoes on gait. *J. Fiber Bioeng. Inform.* **7**, 1–10 (2009).

[29] Gundogdu, Z. Relationship between BMI and blood pressure in girls and boys. *Public Health Nutr.* **11**(10), 1085–1088 (2008). 18426632 10.1017/S1368980008002280

[30] Litvin, F. B. Integrated influence of environmental factors on the state of microcirculation. *Hum. Physiol.* **36**(6), 691–699 (2010). 21254610

[31] Okuno, T. et al. Effects of caffeine on microcirculation of the human ocular fundus. *Jpn. J. Ophthalmol.* **46**(2), 170–176 (2002). 12062222 10.1016/s0021-5155(01)00498-1

[32] Hans, F. J. et al. Nicotine increases microvascular blood flow and flow velocity in three groups of brain areas. *Am. J. Physiol. Heart Circul. Physiol.* **265**(6), H2142–H2150 (1993). 10.1152/ajpheart.1993.265.6.H2142 8285254

[33] Piercy, K. L. et al. The physical activity guidelines for Americans. *Jama* **320**(19), 2020–2028 (2018). 30418471 10.1001/jama.2018.14854 PMC9582631

[34] Colberg, S. R. et al. Physical activity/exercise and diabetes: A position statement of the American Diabetes Association. *Diabetes Care* **39**(11), 2065–2079 (2016). 27926890 10.2337/dc16-1728 PMC6908414

[35] Chao, C. Y. & Cheing, G. L. Microvascular dysfunction in diabetic foot disease and ulceration. *Diabetes Metab. Res. Rev.* **25**(7), 604–614 (2009). 19681035 10.1002/dmrr.1004

[36] Jan, Y. K. et al. Effect of viscoelastic properties of plantar soft tissues on plantar pressures at the first metatarsal head in diabetics with peripheral neuropathy. *Physiol. Meas.* **34**(1), 53–66 (2013). 23248175 10.1088/0967-3334/34/1/53

[37] Lung, C. W. et al. Quantifying dynamic changes in plantar pressure gradient in diabetics with peripheral neuropathy. *Front. Bioeng. Biotechnol.* **4**, 54 (2016). 27486576 10.3389/fbioe.2016.00054 PMC4949238

[38] Hu, H.-F. et al. Combining laser-Doppler flowmetry measurements with spectral analysis to study different microcirculatory effects in human prediabetic and diabetic subjects. *Lasers Med. Sci.* **32**(2), 327–334 (2017). 27928688 10.1007/s10103-016-2117-2

[39] Julious, S. A. Sample size of 12 per group rule of thumb for a pilot study. *Pharm. Stat.* **4**(4), 287–291 (2005).

[40] Newton, D. J. et al. Pilot study of the effects of local pressure on microvascular function in the diabetic foot. *Diabet. Med.* **22**(11), 1487–1491 (2005). 16241911 10.1111/j.1464-5491.2005.01659.x

[41] Melia, G. et al. Insoles of uniform softer material reduced plantar pressure compared to dual-material insoles during regular and loaded gait. *Appl. Ergon.* **91**, 103298 (2021). 33157384 10.1016/j.apergo.2020.103298

[42] Jafarzadeh, M., Tavakoli Golpayegani, A. & Tabatabai Ghomsheh, F. Effect of offloading plantar pressure on peak pressure in ten plantar regions and gait speed in men with diabetes and active diabetic foot ulcers, and healthy men. *Sci. J. Rehabil. Med.* **11**(6), 864–877 (2023).

[43] Lowry, D. et al. The difference between the healing and the nonhealing diabetic foot ulcer: A review of the role of the microcirculation. *J. Diabetes Sci. Technol.* **11**(5), 914–923 (2017). 27390224 10.1177/1932296816658054 PMC5950979

[44] Jeong, D. et al. Application of extracorporeal shockwave therapy to improve microcirculation in diabetic foot ulcers: A prospective study. *Medicine* **102**(11), e33310 (2023). 36930075 10.1097/MD.0000000000033310 PMC10019234

[45] Rayman, G. et al. Impaired microvascular hyperaemic response to minor skin trauma in type I diabetes. *Br. Med. J.* **292**(6531), 1295–1298 (1986). 2939920 10.1136/bmj.292.6531.1295 PMC1340309

[46] Jörneskog, G., Brismar, K. & Fagrell, B. Skin capillary circulation severely impaired in toes of patients with IDDM, with and without late diabetic complications. *Diabetologia* **38**(4), 474–480 (1995). 7796989 10.1007/BF00410286

[47] Balasubramanian, G. V., Chockalingam, N. & Naemi, R. The role of cutaneous microcirculatory responses in tissue injury, inflammation and repair at the foot in diabetes. *Front. Bioeng. Biotechnol.* **9**, 753 (2021). 10.3389/fbioe.2021.732753 PMC8476833 34595160

[48] Gerovasili, V. et al. Physical exercise improves the peripheral microcirculation of patients with chronic heart failure. *J. Cardiopulm. Rehabil. Prev.* **29**(6), 385–391 (2009). 19770806 10.1097/HCR.0b013e3181b4ca4e

[49] Reddy, P. N. et al. Walking cadence affects rate of plantar foot temperature change but not final temperature in younger and older adults. *Gait Posture* **52**, 272–279 (2017). 28012341 10.1016/j.gaitpost.2016.12.008

[50] Duan, Y. et al. A promising method for reducing the incidence of diabetic foot ulcers: Regulating foot temperature during walking. *Med. Hypotheses* **183**, 111268 (2024).

[51] Lohman, E. B. 3rd. et al. A comparison of the effect of a variety of thermal and vibratory modalities on skin temperature and blood flow in healthy volunteers. *Med. Sci. Monit.* **17**(9), 72–81 (2011). 10.12659/MSM.881921 PMC3560507 21873956

[52] Ren, W. et al. Effect of different thermal stimuli on improving microcirculation in the contralateral foot. *BioMed. Eng. Online* **20**(1), 14 (2021). 33531012 10.1186/s12938-021-00849-9 PMC7856788

[53] Waltz, X. et al. Delayed beneficial effect of acute exercise on red blood cell aggregate strength in patients with sickle cell anemia. *Clin. Hemorheol. Microcirc.* **52**, 15–26 (2012). 22414551 10.3233/CH-2012-1540

[54] Karaküçük, Y. et al. Evaluation of the effect of high-intensity interval training on macular microcirculation via swept-source optical coherence tomography angiography in young football players. *Indian J. Ophthalmol.* **69**(9), 2334–2339 (2021). 34427215 10.4103/ijo.IJO_3079_20 PMC8544043

[55] Witana, C. P., Feng, J. & Goonetilleke, R. S. Dimensional differences for evaluating the quality of footwear fit. *Ergonomics* **47**(12), 1301–1317 (2004). 15370849 10.1080/00140130410001712645

[56] Che, H., Nigg, B. M. & de Koning, J. Relationship between plantar pressure distribution under the foot and insole comfort. *Clin. Biomech.* **9**(6), 335–341 (1994). 10.1016/0268-0033(94)90062-0 23916351

